# Large-Area Oxidized Phosphorene Nanoflakes Obtained by Electrospray for Energy-Harvesting Applications

**DOI:** 10.1021/acsanm.0c03465

**Published:** 2021-03-29

**Authors:** Salvatore Moschetto, Margherita Bolognesi, Federico Prescimone, Marco Brucale, Alessio Mezzi, Luca Ortolani, Maria Caporali, Pasqualantonio Pingue, Manuel Serrano-Ruiz, Dario Pisignano, Maurizio Peruzzini, Luana Persano, Stefano Toffanin

**Affiliations:** † Istituto per lo Studio dei Materiali Nanostrutturati (ISMN)—Consiglio Nazionale delle Ricerche (CNR), Via P. Gobetti 101, 40129 Bologna, Italy; ‡ Istituto per lo Studio dei Materiali Nanostrutturati (ISMN)—Consiglio Nazionale delle Ricerche (CNR), P.O. Box 10, Monterotondo Scalo, I-00016 Rome, Italy; § Istituto per la microelettronica e microsistemi (IMM)—Consiglio Nazionale delle Ricerche (CNR), Via P. Gobetti 101, 40129 Bologna, Italy; ∥ Istituto di Chimica dei Composti Organometallici (ICCOM)—Consiglio Nazionale delle Ricerche (CNR), Via Madonna del Piano 10, 50019 Sesto Fiorentino, Florence, Italy; ⊥ Laboratorio NEST, Scuola Normale Superiore and Istituto Nanoscienze—Consiglio Nazionale delle Ricerche (CNR), Piazza San Silvestro 12, I-56127 Pisa, Italy; # Dipartimento di Fisica, Università di Pisa, Largo B. Pontecorvo 3, I-56127 Pisa, Italy

**Keywords:** phosphorene, bidimensional material, large-area, electrospray, electroresponsive, actuators

## Abstract

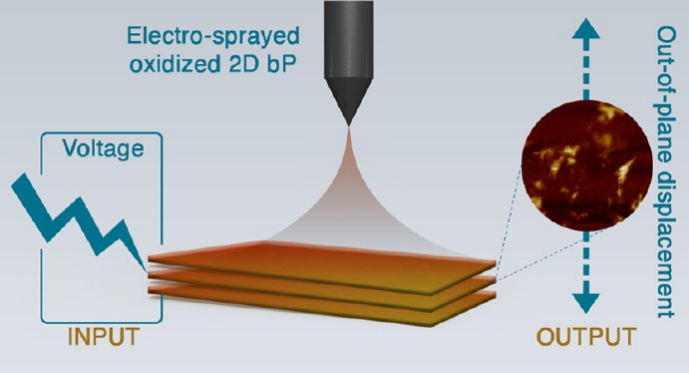

Bidimensional (2D) materials are nowadays being developed as outstanding candidates for electronic and optoelectronic components and devices. Targeted applications include sensing, energy conversion, and storage. Phosphorene is one of the most promising systems in this context, but its high reactivity under atmospheric conditions and its small-area/lab-scale deposition techniques have hampered the introduction of this material in real-world applications so far. However, phosphorene oxides in the form of low-dimensional structures (2D PO*_x_*) should behave as an electroresponsive material according to recent theoretical studies. In the present work, we introduce electrospraying for the deposition of stoichiometric and large-area 2D PO*_x_* nanoflakes starting from a suspension of liquid-phase-exfoliated phosphorene. We obtained 2D PO*_x_* nanostructures with a mean surface area two orders of magnitude larger than phosphorene structures obtained with standard mechanical and liquid exfoliation techniques. X-ray spectroscopy and high-resolution electron microscopy confirmed the P_2_O_5_-like crystallographic structure of the electrosprayed flakes. Finally, we experimentally demonstrated for the first time the electromechanical responsivity of the 2D P_2_O_5_ nanoflakes, through piezoresponse force microscopy (PFM). This work sheds light on the possible implementation of phosphorus oxide-based 2D nanomaterials in the value chain of fabrication and engineering of devices, which might be easily scaled up for energy-harvesting/conversion applications.

## Introduction

In the last decade, the scientific community has focused its attention to bidimensional (2D) nanomaterials, considering this class of systems as highly effective and versatile building blocks for the development of new-generation electronic/optoelectronic devices.^1,2^ Graphene is surely the most studied within 2D materials, but there has been an increasing interest also in the research on new 2D systems, especially semiconducting ones such as transition-metal dichalcogenides (TMDCs) and 2D xenes, i.e., silicene, phosphorene, and borophene.^3^ Phosphorene (or 2D bP) is an atomically thin 2D material that can be obtained from the exfoliation of layered bulk black phosphorus (bP). It shows a lot of interesting properties, such as a direct band gap dependent on the number of atomic layers (0.3–2 eV),^4^ high charge mobility (10^3^ cm^2^/V s),^5^ and high photoluminescence quantum yield.^6^

The high reactivity of phosphorene when exposed to air^7^ is surely one of the most difficult challenges to face to easily use this nanomaterial for large-scale applications.^8^ To protect the material from oxygen, different approaches have been developed, including the direct blending of the phosphorene flakes into a polymeric matrix,^9,10^ or the deposition of a protective/passivating layer,^11,12^ as shown in our previous works.^13,14^ However, the burial of phosphorene into matrices or under protective layers lengthens and complicates process protocol regardless of the desired technological application, from electronic and optoelectronic devices to energy storage and conversion, to chemical sensing.

Otherwise, one could take advantage of the nontrivial properties of low-dimensional phosphorene oxides, or 2D PO*_x_*. 2D PO*_x_* have recently attracted the community for their potential application in electronic, optoelectronic, and energy-harvesting fields, in general.

Ab initio calculations have revealed that the electronic structure of 2D PO*_x_* is strongly dependent on the oxygen concentration, showing a band gap ranging from 4 to 10 eV and an exciton binding energy (*E*_b_) ranging from 1.4 to 3.0 eV, when passing from a low to a high oxygen content (from P_4_O_2_ to P_4_O_10_).^15^ Consequently, both optical and optoelectronic properties in these materials strongly depend on the content/coverage of oxygen. Besides the electronic structure, another recent theoretical study reports that the loss of the mirror symmetry in 2D PO*_x_* nanostructures, compared to pristine phosphorene, leads to a reduction of the phonon lifetime and consequently to great reduction of thermal conductivity. These characteristics make 2D PO*_x_* nanostructures promising candidates for low-dimensional thermoelectric devices.^16^ In addition, thanks again to its noncentrosymmetric structure, recent theoretical calculations^17,18^ predicted that 2D PO*_x_* should show piezoelectric properties. Piezoelectricity, which is the ability to reversibly convert mechanical energy into electrical energy, is a property shared between many materials with a noncentrosymmetric crystalline structure, such as many metal oxides^19^ and metal dichalcogenides.^20,21^ Also, 2D materials such as transition-metal dichalcogenides (TMDCs) or 2D xenes exhibit enhanced piezoelectric properties^19^ with respect to bulk piezoelectric materials. Thus, piezoelectric 2D materials are ideal candidates for applications such as nanosensors,^22,23^ actuators,^24,25^ and energy harvesters,^26−29^ which can operate without an external power supply for sensing, data processing, and data transmission, as highly desired for the Internet-of-Things (IoT).

Here, we introduce an electrospraying technique as an innovative and effective method for the deposition of large-area 2D PO*_x_* nanostructures, which exhibit electromechanical response. The electrospray process, which is based on the application of an electric voltage bias (i.e., tens of kilovolt) between a liquid solution and a collector, has been recently introduced as a scalable method for the deposition of large-area electrodes for applications in solar cells^30^ and supercapacitors.^31^ Once the applied electric field overcomes the surface tension of an element of fluid formed at the termination of an electrified extruder, in fact, charged droplets are formed and sprayed, namely, accelerating toward the collector. Here, the electrospray process was applied to a dispersion of phosphorene (2D bP) flakes in dimethylsulfoxide (DMSO), leading to the formation of crystalline 2D PO*_x_* structures, with an aspect ratio as high as 10 000 (thickness vs lateral dimensions). X-ray photoelectron spectroscopy (XPS), energy-dispersive X-ray analysis (EDX), and transmission electron microscopy (TEM) are used to analyze the composition and the crystalline structure of the deposited 2D nanomaterial. Morphological and mechanical studies are carried out by optical microscopy and atomic force microscopy (AFM). In addition, for the first time, the electroresponsive properties of the deposited 2D nanomaterial are experimentally demonstrated through piezoresponse force microscopy (PFM), showing that the material responds to external electric fields by displaying both vertical and torsional displacements.

The electroresponsive features of the studied 2D PO*_x_* nanostructures make them particularly attractive for energy-harvesting/conversion applications.

## Experimental Section

### bP Synthesis

bP was synthesized according to the method published by Lange, as previously reported by our group.^13,14^ Red phosphorus is used as P-source, and Au, Sn, and SnI_4_ are used as mineralizing agents and catalysts. The mixture is heated in an evacuated quartz vial at temperature *T* = 650 °C for 3 days^32,33^ and then cooled very slowly at room temperature.

### Liquid-Phase Exfoliation

Liquid-phase exfoliation of bP has been extensively studied during the last years. Solvents such as dimethylsulfoxide (DMSO) and dimethylformamide (DMF), which both show a high dielectric constant and high surface tension, have been largely used.^34^ Molecular dynamics simulations have shown that the cohesion energy between the solvent molecules and the bP layers is of great importance, explaining why DMSO, which has a strong affinity with phosphorene, ensures great stability of the dispersion.^35^

In this work, to a suspension of bP microcrystals (5.0 mg) in 5.0 mL of DMSO, deoxygenated distilled water (3–5 μL) was added and the mixture was sonicated for 5 days in the dark keeping the bath temperature at *T* = 19 °C. The obtained 2D bP suspension was either used as it is or after centrifugation for 1 h at 4000 rpm and after collection of the supernatant fraction, for the deposition through electrospray.

### Mechanical Exfoliation

Mechanically exfoliated 2D bP was produced as a reference sample. A bulk bP crystal was mechanically exfoliated in a controlled atmosphere with the scotch tape method (35 times folding). 2D bP flakes obtained in this way were then transferred onto cleaned silicon substrates.

### Deposition of 2D PO*_x_* by Electrospraying

Electrospraying has been recently applied also for the deposition of a variety of large-area 2D materials, including graphene 2D and three-dimensional (3D) structures^31^ and GO.

Here, the whole process was conducted in air and in the absence of heating. The 2D bP suspension obtained from the liquid-phase exfoliation whose concentration is 1 mg/mL (see the description above) was pumped through a syringe into a stainless steel nozzle (inner diameter, 200 μm) at a speed 0.5–1.0 mL/h, by applying a voltage of 11–20 kV between the nozzle and a metal support at a distance of 15 cm (where the deposition occurs).

### Deposition of the Suspension onto Silicon Substrate Previously Coated with 40 nm of Gold

To estimate the total amount of phosphorene oxide obtained by a single electrospray deposition of the 2D bP suspension, we might take into account (i) a reduction of about 25% of the starting concentration (1 mg/mL) of the suspension after centrifugation at 5000 rpm^36^ and (ii) that in a single deposition process of 5 min, the solution used is 0.083 mL. In this scenario, an amount of 0.06 mg of phosphorene oxide is obtained by a single deposition process, which corresponds to a deposition rate of 0.73 mg/h.

### X-ray Photoelectron Spectroscopy (XPS)

XPS analysis was carried out using an ESCALAB 250 Xi, equipped with a monochromatic X-ray source (Al Kα = 148.6 eV) and 6 Channeltron as the detection system. All samples were fixed to a steel sample holder by a metallic clip. The spectra were recorded at 50 eV pass energy at a pressure in the analysis chamber of about 10^–10^ mbar. The BE scale was calibrated positioning the adventitious carbon contribution at BE = 285.0 eV. All data were collected and processed using the Avantage v5.9 software. A signal from a localized spot of 50 μm^2^ of lateral size has been used to analyze a homogeneous area of the selected sample.

### AFM

Topographic AFM of 2D bP and PO*_x_* flakes were performed in PeakForce Tapping mode using Bruker SNL-A probes with a nominal spring constant of 0.35 N/m (Bruker) on a Bruker Multimode 8 microscope equipped with a Nanoscope V controller and a type JV piezoelectric scanner. Force/distance curves were recorded using the same setup at a maximum applied load of 10 nN. The deflection sensitivity of each probe was determined on a silicon oxide surface, and its spring constant was measured with the thermal noise method. All AFM measurements were performed in an atmospheric hood under a gentle flow of either dry nitrogen or air (∼50% humidity) to, respectively, inhibit or promote oxidation. Raw images were processed with Gwyddion^37^ v2.53. Force/distance curves were analyzed with Hooke^38^ rev213.

### PFM Studies

PFM measurements were performed on PO flakes electrosprayed onto silicon substrates, previously coated with 40 nm of gold. A Bruker Dimension Icon system equipped with a Nanoscope V controller was employed as the SPM system. Measurements were carried out in peak force mode, using a platinum–iridium-coated tip with a nominal radius of 20 nm and an elastic constant of 2.8 N/m.

### Transmission Electron Microscopy

Transmission electron microscopy (TEM) was carried out using a Thermo Fischer Tecnai F20 microscope, operated at a 120 kV accelerating voltage to reduce beam damage during observations. Samples from liquid-phase exfoliation were prepared by simple drop-casting of the solutions over a conventional holey-carbon TEM grid and immediately loaded in the microscope to prevent long-term air exposure. Samples of electrosprayed 2D PO*_x_* were prepared by securing a conventional copper TEM grid, covered with a holey-carbon film, over a highly conductive silicon substrate using a drop of conductive silver paint. The electrospraying deposition procedure as described above was performed and the grid is later released from the substrate and loaded inside the microscope for characterization.

### Scanning Electron Microscopy (SEM) and EDX

**Scheme 1:**
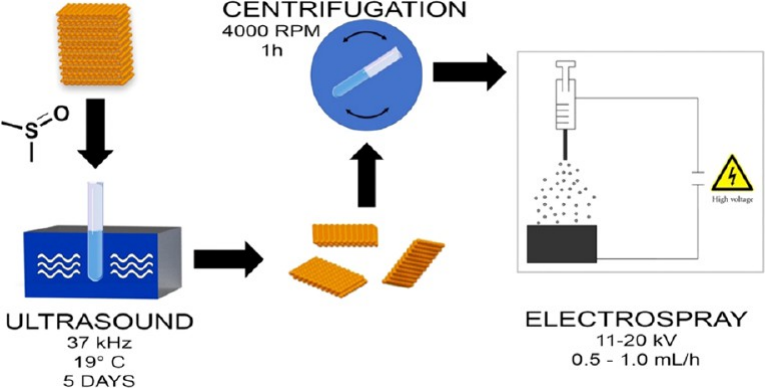
Schematic Illustration of the Deposition of 2D PO*_x_* Flakes Using the Electrospraying Technique, Starting from Liquid-Phase-Exfoliated bP

Scanning electron microscopy (SEM) was performed with an FEI Nova NanoSEM 450 system, equipped with an energy-dispersive X-ray (EDX) detector and operating at acceleration voltages of 5–10 kV (Scheme 1).

## Results and Discussion

### Morphological Characterization

**Figure 1:**
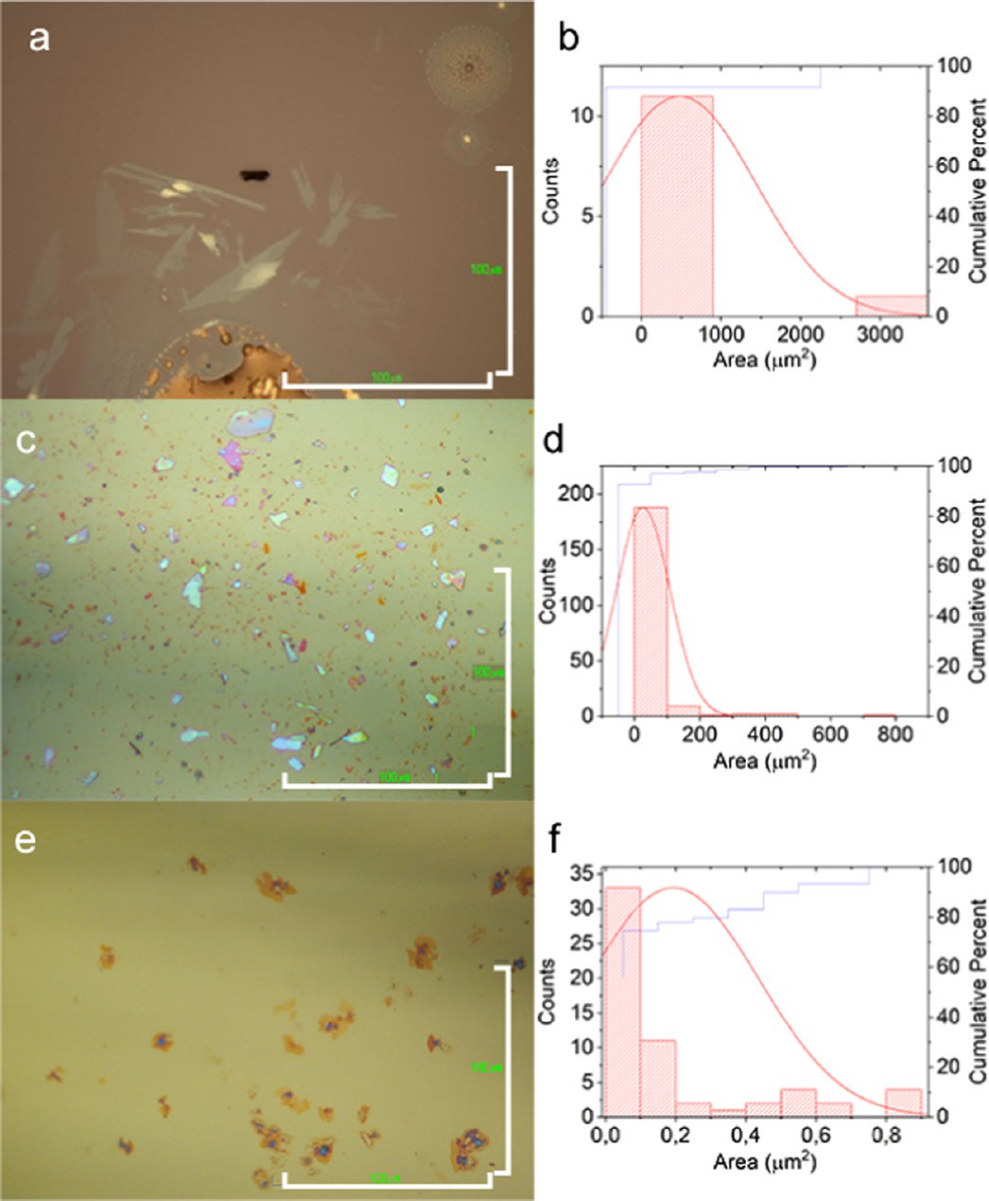
Typical optical micrographs of samples of: (a) 2D structures deposited by electrospraying and (b) relative surface area distribution; (c) 2D bP flakes obtained from the mechanical exfoliation of bP and (d) relative surface area distribution; (e) 2D bP obtained by drop-casting a suspension of liquid-phase-exfoliated bP and (f) relative surface area distribution. Scale bar: 100 μm.

Given the affinity of bP to oxygen, by performing the whole electrospraying process in air, we aimed at depositing a material containing oxidized forms of bP. As the first characterization of the deposited material, we used optical microscopy to analyze the size distribution and the aspect ratio, together with the possible presence of solvent impurities. Optical images of flakes deposited by electrospray were compared with 2D bP samples prepared through liquid-phase exfoliation and oxygen-free mechanical exfoliation of bP. Optical microscopy highlights that the largest elongated solid structures are generally observed closer to big drops of solvent (Figure 1a). In particular, the observed structures have sharp edges and lateral dimensions that range from tens to hundreds of μm^2^, with a very low and homogeneous optical contrast, thus indicating a crystalline 2D nature of the material deposited. It is worth noticing that, unlike previous reports on MXene electrosprayed flaxes,^39^ where crumpled 3D morphologies of the flakes are reported, here, the use of a high-boiling-point solvent (DMSO, boiling point: 189 °C) could enable a more gentle deposition of the flakes onto the collector, thus avoiding the fragmentation and wrinkling processes, which might be associated with a fast evaporation of the solvent during the time of flight.

On the contrary, the optical images of mechanically exfoliated bP (Figure 1c) or liquid-phase-exfoliated bP (Figure 1e) indicate that much smaller crystalline flakes are obtained with these two techniques. Moreover, mechanical and liquid-phase exfoliation produces crystalline flakes with a wide distribution of lateral sizes and thicknesses (evaluated through optical contrast^13^).^40^ For a quantitative comparison, the mean surface areas of the 2D flakes deposited by electrospraying, mechanical exfoliation, and liquid-phase exfoliation were calculated, resulting in 480, 26.3, and 0.2 μm^2^, respectively (Figure 1b,d,f). These values clearly highlight the effectiveness of the proposed method for the deposition of large-area 2D structures.

**Figure 2:**
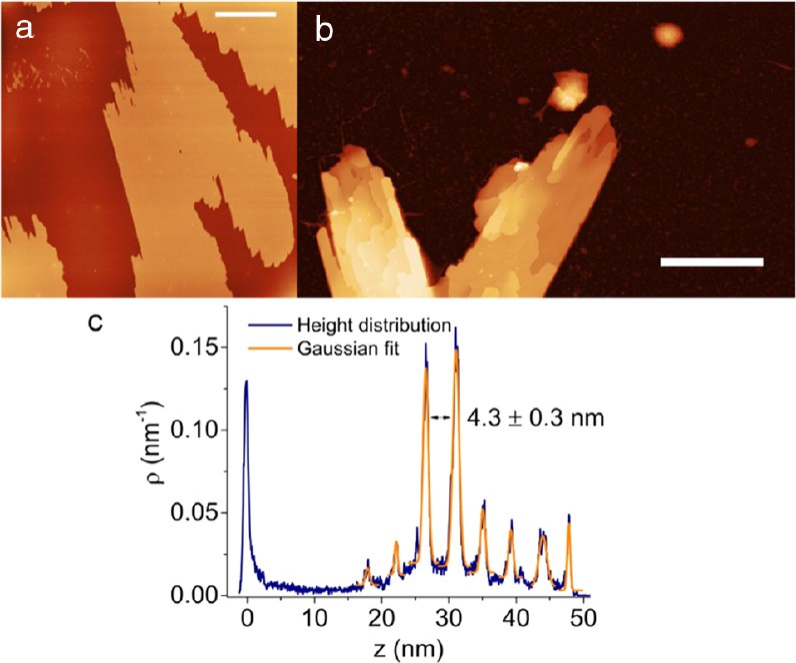
AFM height image of (a) a single-layer flake and (b) a multilayered flake, obtained through electrospray. (c) Height distribution of the multilayered flake as extracted from image (b). Scale bar: (a) 30 μm; (b) 5 μm. Vertical scales: (a) −10–20 nm; (b) −5–55 nm.

The morphology of the 2D structures obtained by electrospraying was further analyzed through AFM. In many regions of each substrate, single-layer 2D structures are found (Figure 2a). The surface of these structures is atomically flat (full width at half-maximum (FWHM) of peak height is <1 nm) and extends over a micrometric area. It should be noted that the uniform thickness of these flakes is fundamental in view of their application in electronics because the electronic properties are strongly thickness-dependent. In addition, the morphology of the single-layer structures is highly stable and remains unchanged after overnight exposure to air, as observed from the AFM image taken on the same surface of the same 2D flake (Figure S1 in the Supporting Information). This is in striking contrast to that typically found for mechanically exfoliated 2D bP, since the first oxidation features are already observed within 1 h after air exposure.^13^

By contrast, the thermal stability of electrosprayed PO*_x_* flakes in inert atmosphere is lower compared to mechanically exfoliated 2D bP. The optical images before and after annealing at 400 °C for 15 min (Figure S2) show the total removal of PO*_x_* flakes from SiO_2_ substrate due to the thermal treatment. Instead, annealing at 200 °C, for the same time (15 min) resulted in only a partial removal of PO*_x_* flakes. We can assume that PO*_x_* flakes are stable at temperatures lower than 200 °C (Figure S3). It is likely that annealing at temperatures higher than 200 °C leads to the complete removal of the PO*_x_* flakes due to their weak adhesion onto the SiO_2_ substrate, which may be further weakened or inhibited by the presence of residual solvent from the electrospray process.

On the contrary, 2D bP flakes obtained by mechanical exfoliation and transferred onto SiO_2_ substrates are thermally stable up to 600 °C. XPS spectra of 2D bP flakes onto SiO_2_ (Figure S4, Table S1) show indeed that the energy of the P 2p signal related to phosphorous remains unaltered before and after the annealing process, revealing the high thermal stability of nonoxidized 2D bP. We can conclude that more favorable interactions probably occur between mechanically exfoliated 2D bP flakes and SiO_2_, compared to electrosprayed PO*_x_* flakes onto SiO_2_.

2D multilayered structures can be also found (Figure 2b). The height distribution graph obtained from the AFM images of the 2D multilayered structure reveals that the layers are equally separated by an average distance of about 4.3 ± 0.3 nm (Figure 2c). This indicates that the electrosprayed 2D material obtained is layered, like 2D bP, but it has a different crystalline structure and composition, since its interlayer distance is different from that in pristine 2D bP (5.28 Å)^41^ and also different from that expected from 2D bP intercalated with solvent molecules (i.e., 5.4 Å).^42^

**Figure 3:**
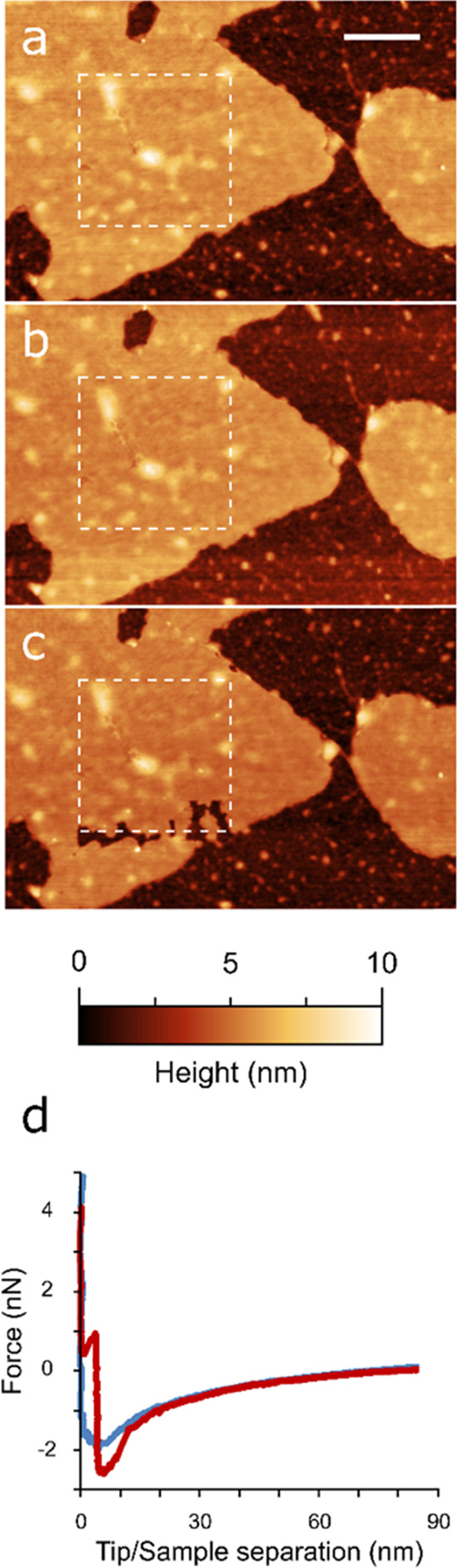
Mechanical response of single electrosprayed flake as observed via AFM. (a–c) Mechanical failures induced by repeatedly scanning the same 1 × 1 μm^2^ area (dashed white lines) at 1 nN applied force in PeakForce mode (512 × 512 lines, 0.5 Hz scan rate). After each successive scan, the flake was then imaged at approximately 250 pN applied force at a lower magnification to appreciate potentially induced damages. Failures are apparent after 51 min of total scanning at higher forces (c). (d) Representative nanoindentation force/separation curves performed on electrosprayed flake (red) or a mechanically exfoliated 2D bP flake (blue). The red trace shows a clear breakthrough event with first contact occurring at 4.7 nm and failure at 3.9 nm. The vast majority (96%) of force curves performed on electrosprayed flakes showed one or more breakthrough events of this type, whereas no sudden mechanical failure “steps” were observed on mechanically exfoliated 2D bP flakes subjected to the same compressive stresses. Scale bar: 250 nm.

Moreover, the repeated AFM scans on the same area of the same flake (Figure 3a) highlighted a larger fragility of the electrosprayed material with respect to the mechanical exfoliated 2D bP. For instance, after 15 min of scanning (Figure 3b), the AFM image reveals a hole in the center of the flake, which widens during further scans (Figure 3c). The majority of force curves recorded on intact regions of single-layer electrosprayed flakes show a sharp breakthrough event of a 4.5 nm thick layer at around 1–3 nN applied force (Figure 3d). In contrast, single- or few-layer pristine 2D bP flakes remain unperturbed over 270 min under the same AFM scanning conditions and do not show layer breakthrough events when subjected to forces up to ∼5 nN (see Figure 3d, blue line). The higher brittleness of the oxidized material seems to further confirm the presence of a material with chemical composition and crystalline habit that are different from 2D bP. In detail, this mechanical behavior is predicted by theoretical calculations recently reported in the literature for phosphorene oxides.^43^

### Compositional, Chemical, and Structural Characterization

To verify the composition of the deposited material, we performed the chemical characterization of the surface of the samples. Due to the heterogeneity of the electrosprayed samples (also related to the presence of solvent droplets onto the substrate and to the subsequent solvent evaporation pattern), XPS, EDX, and TEM analyses were performed only on selected areas of the substrate, where the 2D structures were observed by optical microscopy.

XPS measurements of electrosprayed samples were compared to those of mechanically exfoliated 2D bP as the reference. In particular, the oxidation state of the phosphorus atoms in the two samples was investigated.

**Figure 4:**
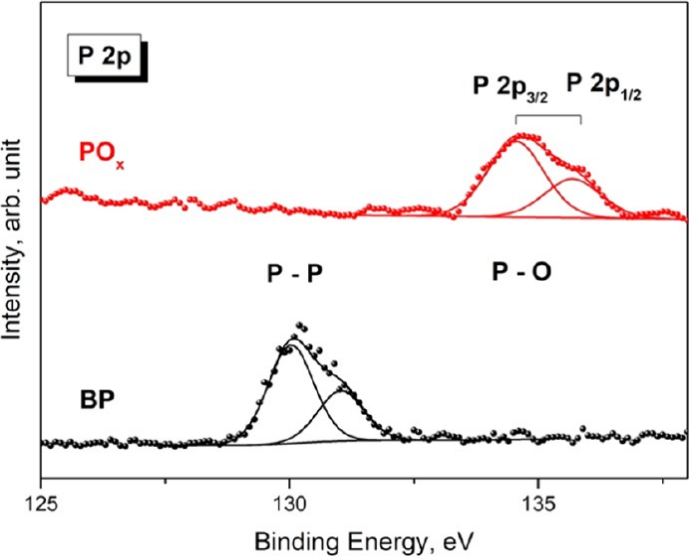
XPS spectra of P 2p acquired on mechanically exfoliated 2D bP and electrosprayed samples.

Figure 4 shows the P 2p spectra of both samples. P 2p peak is characterized by the typical doublet due to the spin–orbit splitting P 2p_3/2_–P 2p_1/2_, separated by Δ(BE) = 1.3 eV. This signal is generally found in the range of binding energy (BE) = 129.0–135.0 eV, depending on the bond in which P is involved. To avoid the plasmon loss of Si 2p that for Si(0) lies in the proximity of the P 2p signal, electrospray and mechanical exfoliations were performed on silicon substrates covered with a thermally grown layer of SiO_2_. The mechanically exfoliated 2D bP sample was characterized by a P 2p_3/2_ peak positioned at BE = 130.0 eV, which is characteristic for P–P bond.^44^ The good quality of 2D bP was also testified by the absence of oxides contribution, generally positioned at higher BE.^45^ In the case of the electrosprayed sample, the P 2p_3/2_ peak was positioned at BE = 134.1 eV, which is typical for P at a higher oxidation state (+5), in the configuration of P_2_O_5_. The absence of a peak positioned at BE = 130.0 eV highlights that none (or a negligible part) of the P atoms in electrosprayed sample are in the 0 oxidation state, such as in BP. These results highlight and confirm that the proposed electrospray deposition is a good method to produce an oxidized phosphorus-based material (2D PO*_x_*).

Other than P signals, the XPS quantitative analysis revealed also the presence of other elements, such as C, O, N, and Si (Table S2). The Si 2p signal was assigned to the substrate because the information depth of the XPS is higher than the thickness of 2D PO*_x_*. The C 1s signal was characterized by two components positioned at BE = 285.0 eV (14.8 atom %) and 286.6 eV (1.6 atom %), assigned to aliphatic carbon and C–O bond, respectively. As regards the O 1s signal, there was a strong contribution of SiO_2_ at 533.1 eV (52.1 atom %), while the contribution of P–O and C–O was found at BE = 531.2 eV (3.6 atom %). Finally, the presence of N was detected, where N 1s signal was positioned at BE = 402.4 eV, assigned to residual NO groups from the sample preparation.

The local analysis through SEM microscopy and EDX spectroscopy confirmed that the signal from oxidized species identified by XPS can be attributed to the electrosprayed material.

**Figure 5:**
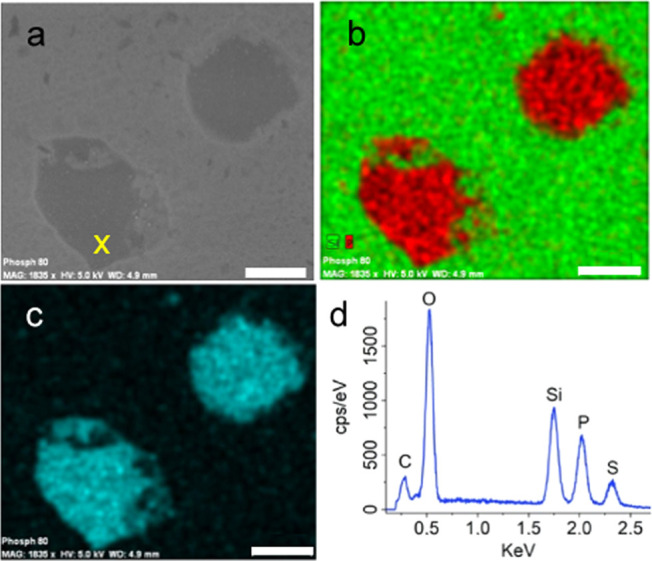
(a) SEM image of 2D PO*_x_* flakes. (b, c) Phosphorus (red), silicon (green), and oxygen (cyan) EDX maps recorded from the 2D PO*_x_* flakes shown in (a). (d) EDX spectrum taken at the point indicated with X in (a). Scale bar: 30 μm.

Figure 5a shows the SEM image of the sample prepared through electrospraying, and Figure 5b shows the corresponding elemental analysis map extrapolated from EDX measures on the same area. The darker areas of the SEM image correspond to phosphorus-rich areas, while on the substrate, only Si and O signals are registered. The EDX spectrum (Figure 5c) registered at the point X of Figure 5a is characterized by P, Si, and S peaks arising, respectively, from (i) the 2D PO*_x_* structure observed, (ii) the substrate, and (iii) the residual DMSO solvent in proximity of the 2D structure.

While the composition of the electrosprayed structures was confirmed through XPS and EDX analyses, TEM was performed to investigate their crystalline features.

**Figure 6:**
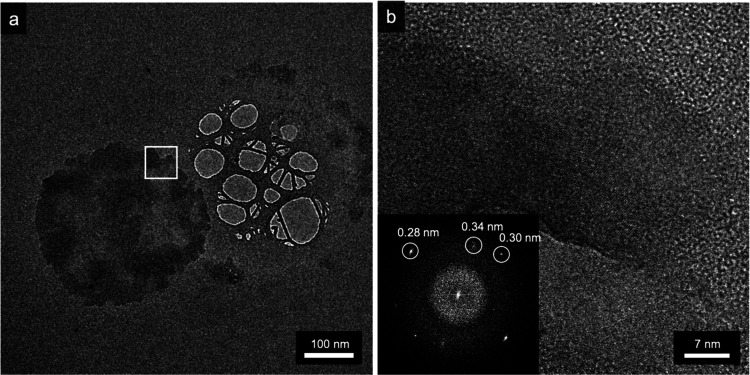
TEM characterization of electrosprayed 2D PO*_x_* material. (a) TEM image of crystal aggregate over the amorphous carbon film of the TEM grid. (b) HREM image of the area highlighted by the white rectangle in (a), showing lattice fringes of the deposited material; (inset) FFT of the HREM image showing reflections corresponding to planes spaced by 0.34, 0.30, and 0.28 nm, compatible with the P_2_O_5_ crystal structure.

Figure 6a shows a PO*_x_* aggregate over the amorphous carbon film of the TEM grid, with a rather regular circular shape, probably originating from a nanodroplet of the spray procedure. It should be revealed that the size and shape of the PO*_x_* flakes deposited through electrospraying are largely dependent on the substrate, explaining why PO*_x_* flakes analyzed by TEM have smaller lateral dimensions and different shapes with respect to the flakes deposited over Si or Si/SO_2_ substrates and analyzed through XPS, AFM, and PFM. The area highlighted in the white rectangle is further magnified in the high-resolution electron microscopy (HREM) image in Figure 6b, where lattice fringes are visible. The fast Fourier transform (FFT) of the image is shown in the inset: the marked spots correspond to periodicities of 0.34, 0.30, and 0.28 nm. While the exact crystal orientation was impossible to be determined, the periodicities shown here are compatible with the crystal structure of P_2_O_5_ found in the literature,^46^ namely, (120) 0.335 nm, (211) 0.305 nm, and (220) 0.283 nm, consistent with the composition found by XPS analysis.

**Figure 7:**
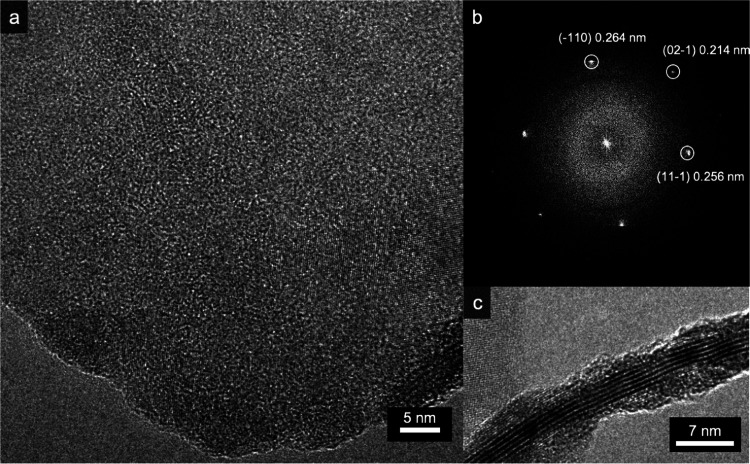
TEM characterization of liquid-phase-exfoliated bP. (a) High-resolution TEM (HRTEM) image of a black phosphorous flake edge. (b) FFT of the image, showing black phosphorous (1̅10), (021̅), and (111̅) reflections. (c) Details of the flake folded edge, showing (002) lattice fringes.

TEM analysis of 2D bP obtained by liquid-phase exfoliation of bulk bP exhibits largely different results from the electrosprayed samples. Figure 7a shows the HREM image of the edge of a bP nanosheet, and Figure 7b displays the corresponding FFT, where reflections compatible with bP crystal structure are highlighted. The flake is oriented with the (112) direction aligned with the viewing direction, showing (1̅10), (021̅), and (111̅) reflections in the FFT. When the flake edge folds over itself, (002) lattice fringes, spaced by 0.525 nm, are visible in the HREM image, as shown in Figure 7c, and the number of stacked layers can be determined by counting the number of fringes visible in the image (six layers, in the case of the shown image).

### Electromechanical Responsivity

**Figure 8:**
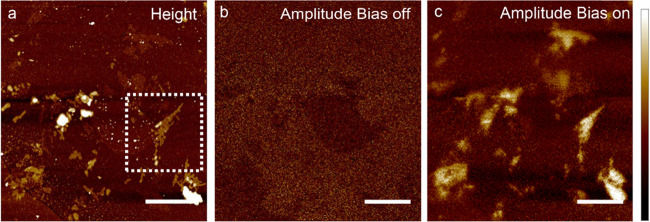
AFM topographic image and the corresponding PFM image at bias voltages 0 V (b) and 5 V (c). Scale bar: 3 μm. Vertical scales: (a) −12–20 nm; (b, c) −17–30 pm.

Piezoresponse force microscopy (PFM) probes the surface mechanical deformations of a material in response to an external voltage applied between an AFM tip and a bottom electrode (usually a conductive substrate underneath the investigated sample). This might unveil the electromechanical functionality of the material at the nanometer and micrometer scales. The applied voltage is *V*_ext_ = *V*_dc_ + *V*_ac_ × cos(ω*t*), where *V*_dc_ is an optional dc offset and *V*_ac_ is the amplitude of the alternated voltage with angular frequency ω. In electromechanically active materials, the surface can deform mechanically in response to the external electric field. By the use of a position-sensitive detector for the cantilevered probe, the PFM technique can track both out-of-plane and in-plane components of the material response at the modulated frequency by lock-in detection, which is generally useful for systems with an arbitrary polarization direction. Electromechanically active materials exhibit a nonzero amplitude PFM signal when an external field is applied. Figure 8a shows a typical AFM image of electrosprayed 2D P_2_O_5_ flakes. The corresponding out-of-plane amplitude images, when no bias is applied to the tip-sample system and upon the application of 5 V, are reported in Figure 8b,c.

**Figure 9:**
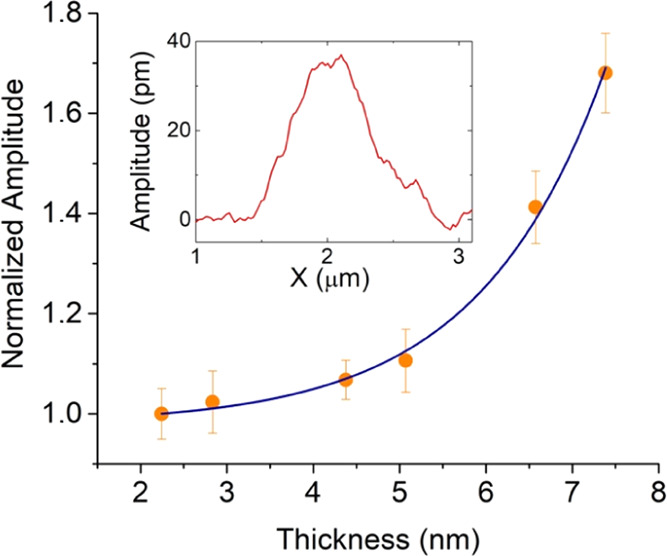
Maximum out-of-plane amplitude intensity vs flake thickness (black dots). The line is a guide to the eye. Inset: Typical out-of-plane amplitude profile.

Micrographs clearly indicate that 2D P_2_O_5_ flakes are sensitive to the applied external field and generate a strain response of the order of tens of picometers. A more in-depth investigation of the electromechanical response of one of the flakes in Figure 8a is reported in the Supporting Information (Figure S5b,c with out-of-plane and in-plane amplitude signals recorded at *V*_dc_ = 0, *V*_ac_ = 5 V, and a frequency of 30 kHz). Out-of-plane and in-plane signals, both as amplitude and phase signals, have a similar spatial distribution that well matches the topography, thus indicating the presence of both vertical and torsional displacements (related to domains sensitive to shear forces), which can be correlated to domain patterns oriented at certain angles with respect to the sample plane. The phase image in Figure S5d indicates an out-of-phase response (i.e., flakes appear dark), corresponding to dipoles oriented toward the bottom electrode. Out-of-plane dipoles are sensitive to the direction of the applied field and exhibit a 180° phase shift (i.e., flakes appear bright) when the external bias is inverted (i.e., the positive bias is applied to the sample and the tip is grounded) as reported in Figure S5e. Note that the amplitude of the out-of-plane signal, some picometers per V (4–5 pm/V), is comparable to the theoretical values reported in the literature for phosphorene oxides.^17^ Moreover, the out-of-plane amplitude intensity increases with the thickness of P_2_O_5_ flakes (Figure 9).

This behavior is in agreement with previous reports on flakes of 2D materials such as sulfides and selenides^47,48^ and can be ascribed to a nonuniform distribution of the electric field in the tip-sample system, especially for materials with low electrical permittivity.^49,50^ The presence and amplitude of the out-of-plane piezoresponse signals and their increment versus thickness, allow us to include electrosprayed 2D P_2_O_5_ in the class of electromechanical responsive materials. Envisaged applications, for which electrosprayed 2D P_2_O_5_ might establish new design rules, include hybrid nanoelectronics/nanogenerator platforms, optomechanical devices, and sensing architectures based on shear or strained components.

## Conclusions

In summary, we demonstrated that the electrospraying technique is a useful tool to effectively deposit an air-stable, phosphorus oxide-based nanomaterial in the 2D form, specifically as 2D P_2_O_5_ nanoflakes as confirmed by the XPS, SEM-EDX, and HRTEM analyses. AFM confirmed that the electrosprayed, large-area P_2_O_5_ nanostructures have an atomically flat surface, a thickness in the nanometer range (single-layered structures), and a periodicity between layers of about 4.3 ± 0.3 nm (few-layer structures), confirming the extremely high surface-to-volume ratio and 2D nature of such P_2_O_5_ structures. AFM also confirmed the higher stability in air and higher mechanical fragility of the 2D P_2_O_5_ nanoflakes with respect to 2D bP. Notably, the lateral size of the electrosprayed 2D P_2_O_5_ flakes (hundreds of μm^2^) is much larger than the average lateral size of 2D bP flakes obtained with standard techniques such as mechanical and liquid-phase exfoliation of bP (tens of μm^2^). Other than the larger lateral size and the improved stability, the electrosprayed 2D P_2_O_5_ flakes showed a clear electromechanical responsivity. PFM was indeed performed onto the electrosprayed 2D P_2_O_5_ flakes to demonstrate their electromechanical response, confirming theoretical predictions from the literature on 2D oxidized phosphorus nanostructures. The electrosprayed 2D P_2_O_5_ flakes show in fact both in-plane signal (tenths of picometers per volt) and out-of-plane signal (some picometers per volt) when subjected to an external electric field.

Overall, the approach herein proposed to deposit a large-area, electromechanical responsive 2D P_2_O_5_ allows us to overcome the process scalability issues and enables the real applicability of phosphorus-based 2D nanomaterials for sensing and various classes of energy-harvesting devices.

## Supporting Information Available

Optical, AFM, PFM, and XPS characterizations of oxidized phosphorene obtained by electrospray deposition (PDF)
