# Supplementary material for: Large-Area Oxidized Phosphorene Nanoflakes Obtained by Electrospray for Energy-Harvesting Applications
Source: ACS Appl Nano Mater. 2021 Mar 29;4(4):3476–85. doi: 10.1021/acsanm.0c03465 (PMC9301623; doi:10.1021/acsanm.0c03465)
Supplement: Supplementary file 1 — an0c03465_si_001.pdf [file an0c03465_si_001.pdf]

Supporting Information

Large-Area Oxidized Phosphorene Nanoflakes Obtained by Electrospray for Energy-Harvesting Applications

Salvatore Moschetto,,† Margherita Bolognesi,† Federico Prescimone,† Marco Brucale,† Alessio Mezzi,§ Luca Ortolani,ǁ Maria Caporali,⊥ Pasqualantonio Pingue,# Manuel Serrano-Ruiz,⊥ Dario Pisignano,#,‡ Maurizio Peruzzini,⊥ Luana Persano,*,# Stefano Toffanin*,†.*

†Istituto per lo Studio dei Materiali Nanostrutturati (ISMN) - Consiglio Nazionale delle Ricerche (CNR), Via P. Gobetti 101, 40129 Bologna, Italy

§Istituto per lo Studio dei Materiali Nanostrutturati (ISMN) - Consiglio Nazionale delle Ricerche (CNR), P.O. Box 10, I-00016 Monterotondo Scalo, Rome, Italy

ǁIstituto per la microelettronica e microsistemi (IMM) - Consiglio Nazionale delle Ricerche (CNR), Via P. Gobetti 101, 40129 Bologna, Italy

⊥Istituto di Chimica dei Composti Organometallici (ICCOM) - Consiglio Nazionale delle Ricerche (CNR), Via Madonna del Piano 10, 50019 Sesto Fiorentino, Florence, Italy

Laboratorio NEST, Scuola Normale Superiore and Istituto Nanoscienze - Consiglio Nazionale delle Ricerche (CNR), Piazza San Silvestro 12, I-56127 Pisa, Italy

‡Dipartimento di Fisica, Università di Pisa, Largo B. Pontecorvo 3, I-56127 Pisa, Italy

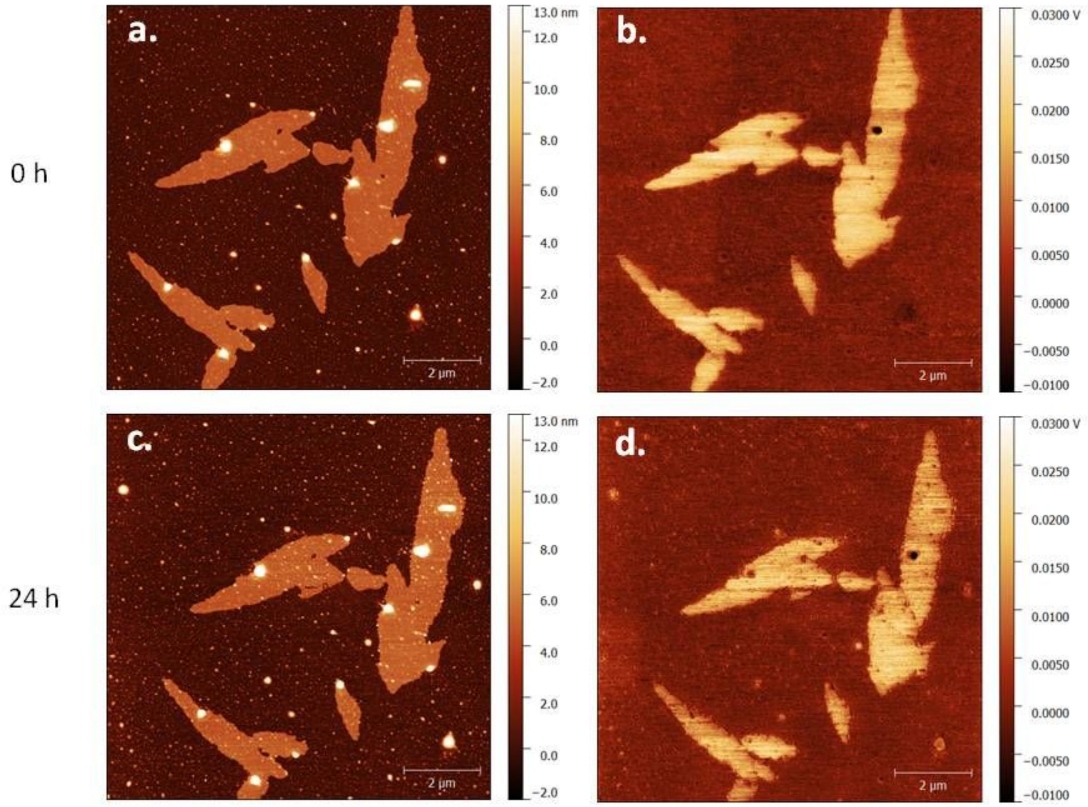

Figure S1. AFM height (a, c) and phase (b, d) images of the same PO_x flakes, immediately after deposition (a, b) and after 24 hours of exposure (b, d) to standard laboratory conditions (approximately 50% humidity and 400 lux of illumination). Both height and phase of investigated monolayer flakes remain unchanged by air exposure, not showing the typical

oxidation features appearing as bubble-like protrusions over bare phosphorene flakes after air exposure[1]. These results confirm the high chemical stability of the PO_x.

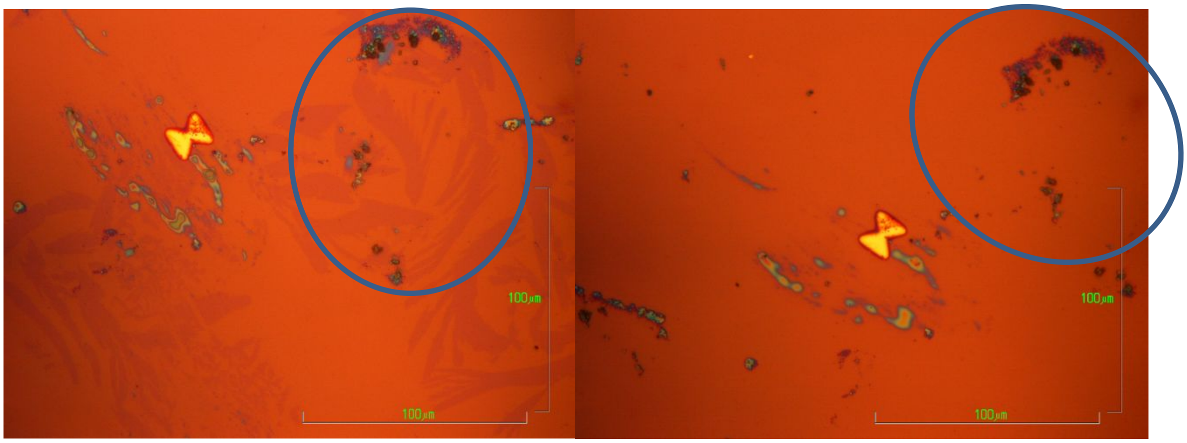

Figure S2. Optical images on the left and on the right show respectively the PO_x flakes before and after the annealing process (400°C, 15 minutes).

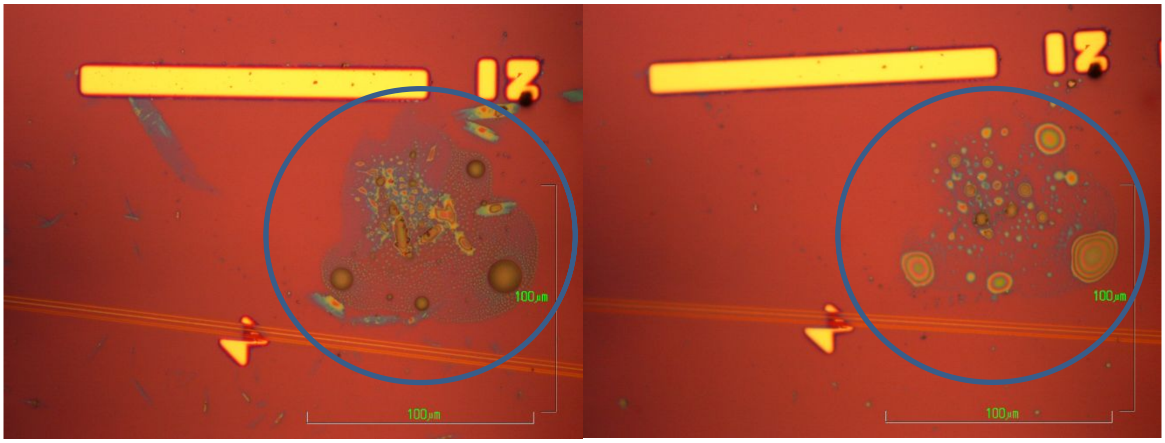

Figure S3. The optical images on the left and on the right show respectively the PO_x flakes before and after the annealing process (200°C, 15 minutes).

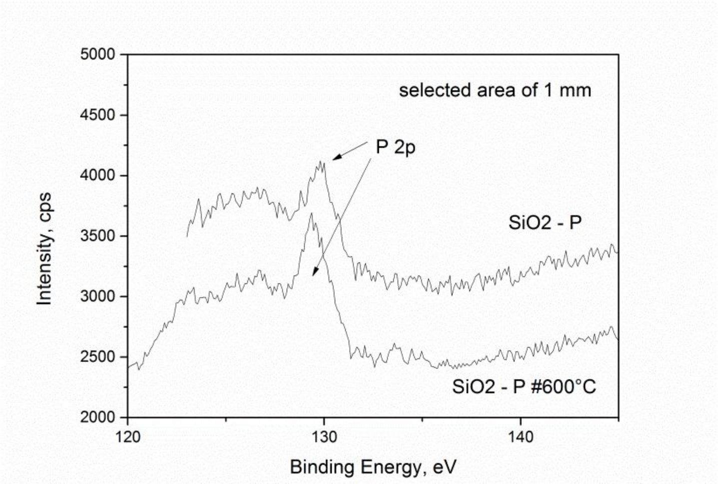

Figure S4. XPS spectra of 2D bP on SiO_2 substrate before and after annealing (600° C, 1 hour).

Table S1. Binding Energy (BE) and atomic concentration (atomic %), acquired in two different point of the 2D bP flakes on Si/SiO_2 substrate.

Signal	Peak BE (eV)	Atomic %	Bond
C1s – 1	285.0	6.0	C – C
O1s	533.1	58.0	SiO_2
P2p	129.9	0.5	Phosphorene
Si2p3	103.6	33.0	SiO_2

Signal	Peak BE (eV)	Atomic %	Bond
C1s	285.0	2.5	C – C
O1s	533.1	61.9	SiO_2
P2p	129.4	0.6	Phosphorene
Si2p3	103.4	35.1	SiO_2

Table S2. XPS signals of P, C, O, N and Si revealed on the electro-sprayed P_2O_5 flakes.

		**C1s (C - C)**	**C1s (C - O)**			**O1s**	**P2p$_{3/2}$ (P - P)**	
**Bulk bP**	BE (eV)	284.7	287.2			532.5	130.0	
	At. %	40.1	11.1			5.6	43.3	
		C1s (C - C)	C1s (C - O)	C1s (-COOH)	O1s (SiO_2)		P2p$_{3/2}$ (P - P)	Si2p$_{3/2}$ (SiO_2)
**exfoliated bP**	BE (eV)	285.0	286.9	289.0	533.1		130.0	103.6
	At. %	6	1.6	1.0	58.0		0.5	33.0
		C1s (C - C)	C1s (C - O)	N1s (NO)	O1s (SiO_2)	O1s (P - O, C - O)	P2p$_{3/2}$ (P - O)	Si2p$_{3/2}$ (SiO_2)
**PO_x**	BE (eV)	285.0	286.6	402.4	533.1	531.2	134.3	103.6
	At. %	14.8	1.6	3.3	52.1	3.6	2.0	22.6

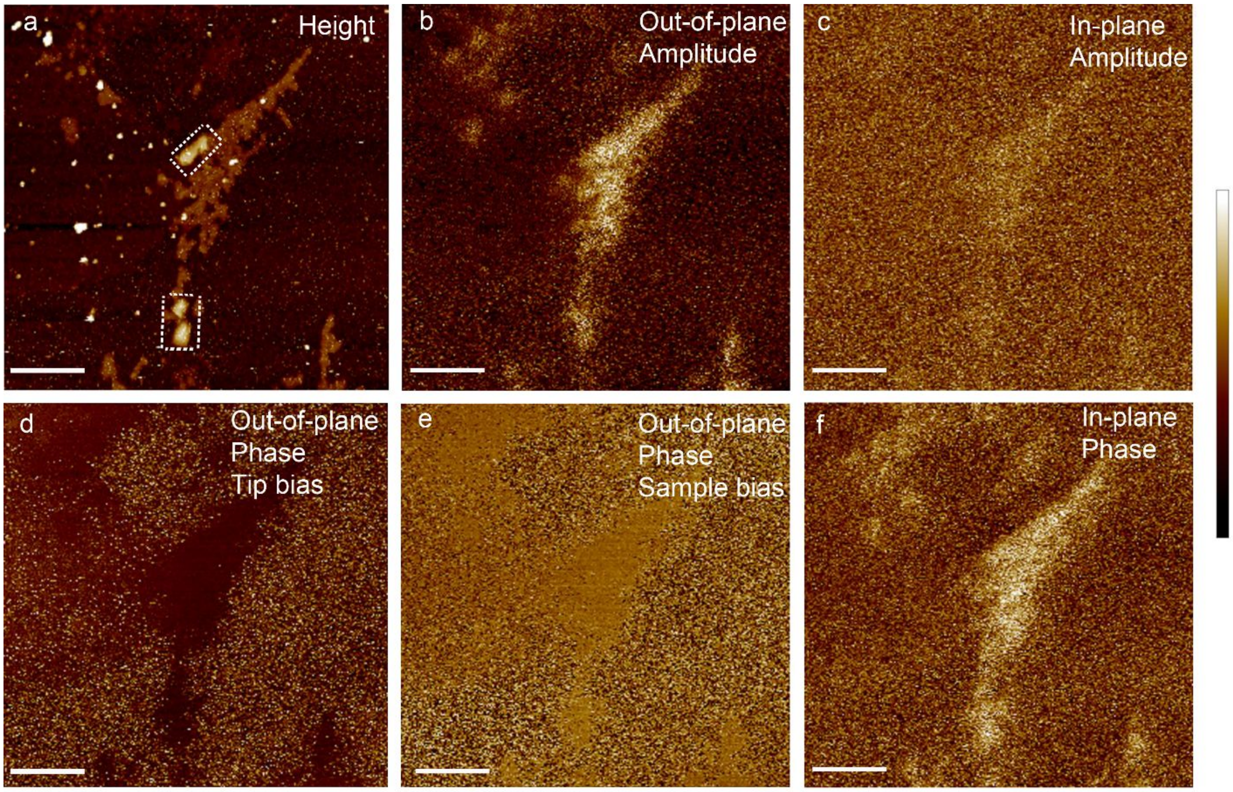

Figure S5. Piezoforce microscopy (PFM) micrographs of P_2O_5 flakes. a) Height image, vertical scale: -8 nm-20 nm. b) Piezoresponse out-of-plane amplitude, vertical scale -20-40pm. c) Piezoresponse in-plane amplitude, vertical scale 7-11 pm. d-e) Piezoresponse out-of-plane phase shift. In d (e) a voltage bias is applied to the tip (sample). f) Piezoresponse in-plane phase image. Scale bars: 1 μm.

(1) Bolognesi, M.; Brucale, M.; Lorenzoni, A.; Prescimone, F.; Moschetto, S.; Korolkov, V. V.; Baldoni, M.; Serrano-Ruiz, M.; Caporali, M.; Mercuri, F.; Besley, E.; Muccini, M.; Peruzzini, M.; Beton, P. H.; Toffanin, S. Epitaxial Multilayers of Alkanes on Two-Dimensional Black Phosphorus as Passivating and Electrically Insulating Nanostructures. *Nanoscale* **2019**, *11* (37), 17252–17261.

Corresponding Authors:

*E-mail: stefano.toffanin@cnr.it

*E-mail: luana.persano@nano.cnr.it

*E-mail: salvatore.moschetto@ismn.cnr.it
